# Cerebral Blood Flow and Aβ-Amyloid Estimates by WARM Analysis of [^11^C]PiB Uptake Distinguish among and between Neurodegenerative Disorders and Aging

**DOI:** 10.3389/fnagi.2016.00321

**Published:** 2017-01-11

**Authors:** Anders B. Rodell, Graeme O’Keefe, Christopher C. Rowe, Victor L. Villemagne, Albert Gjedde

**Affiliations:** ^1^Centre for Clinical Research, University of Queensland, BrisbaneQLD, Australia; ^2^Department of Nuclear Medicine & PET-Centre, Aarhus University HospitalAarhus, Denmark; ^3^Department of Molecular Imaging and Therapy, Centre for PET, Austin Health, HeidelbergVIC, Australia; ^4^Department of Neuroscience and Pharmacology, University of CopenhagenCopenhagen, Denmark; ^5^Department of Neurology and Neurosurgery, McGill University, MontréalQC, Canada; ^6^Division of Nuclear Medicine, Department of Radiology and Radiological Science, Johns Hopkins University, BaltimoreMD, USA; ^7^Neurosciences Research Center, Tabriz University of Medical SciencesTabriz, Iran; ^8^Department of Clinical Medicine – Department of Nuclear Medicine, University of Southern DenmarkOdense, Denmark

**Keywords:** Alzheimer’s disease, CBF, amyloid-β, [^11^C] PiB, flow normalization, parametric imaging

## Abstract

**Background:** We report results of the novel Washout Allometric Reference Method (WARM) that uses estimates of cerebral blood flow and amyloid load from the same [^11^C]Pittsburgh Compound B ([^11^C]PiB) retention maps in brain to distinguish between patients with different forms dementia, including Alzheimer’s disease, and healthy volunteers. The method introduces two approaches to the identification of brain pathology related to amyloid accumulation, (1) a novel analysis of amyloid binding based on the late washout of the tracer from brain tissue, and (2) the simultaneous estimation of absolute cerebral blood flow indices (sCBF) from the early accumulation of the tracer in brain tissue.

**Objective:** We tested the hypothesis that a change of cerebral blood flow is correlated with the degree of tracer [^11^C]PiB retention, reflecting dendritic spine pathology and consequent inhibition of brain energy metabolism and reduction of blood flow by neurovascular coupling in neurodegenerative disorders, including Alzheimer’s disease.

**Methods:** Previously reported images of [^11^C]PiB retention in brain of 29 subjects with cognitive impairment or dementia [16 Alzheimer’s Disease (AD), eight subjects with dementia with Lewy bodies (DLB), five patients with frontotemporal lobar degeneration (FTLD), five patients with mild cognitive impairment, and 29 age-matched healthy control subjects (HC)], underwent analysis of PiB delivery and retention by means of WARM for quantitation of [^11^C]PiB’s binding potentials (*BP*_ND_) and correlated surrogate cerebral blood flow (sCBF) estimates, based on the [^11^C]PiB images, compared to estimates by conventional Standard Uptake Value Ratio (SUVR) of [^11^C]PiB retention with cerebellum gray matter as reference. Receiver Operating Characteristics (ROC) revealed the power of discrimination among estimates.

**Results:** For AD, the discriminatory power of [^11^C]PiB binding potential (*BP*_ND_) by WARM exceeded the power of SUVR that in turn exceeded the power of sCBF estimates. Differences of [^11^C]PiB binding and sCBF measures between AD and HC both were highly significant (*p* < 0.001). For all the dementia groups as a whole, sCBF estimates revealed the greatest discrimination between the patient and HC groups. WARM resolves a major issue of amyloid load quantification with [^11^C]PiB in human brain by determining absolute sCBF and amyloid load measures from the same images. The two parameter approach provides key discriminary information in AD for which [^11^C]PiB traditionally is used, as well as for the distinct flow deficits in FTLD, and the marked parietal and occipital lobe flow deficits in DLB.

**Conclusion:** We conclude that WARM yields estimates of two important variables that together discriminate among patients with dementia, including AD, and healthy volunteers, with ROC that are superior to conventional methods of analysis. The distinction between estimates of flow and amyloid load from the same dynamic emission tomograms provides valuable pathogenetic information.

## Introduction

Two properties characterize different forms of dementia, i.e., the degree of functional decline, and the specific pathology leading to the functional decline. In humans, dementia is defined by deficiency in at least two cognitive domains. The functional decline, in turn, is linked to disturbances of brain energy metabolism and blood flow, with changes of glucose consumption and cerebral blood flow held to occur in parallel. However, the pathological processes underlying the different types of dementia in humans are poorly understood, in part because specific evidence of the pathology and quantitative measures of brain energy metabolism and cerebral blood are not always available for simultaneous scrutiny, not the least when the evidence derives from different methods that typically are not applied at the same time.

The loss of functional integrity associated with dementia is reflected in impairments of brain energy metabolism and blood flow that commonly are determined by mapping of one or more indices of brain function, including maps of brain glucose consumption obtained by means of positron emission tomography (PET; Rodell et al., 2013), or maps of cerebral blood flow obtained by any one of a number of PET and magnetic resonance imaging (MRI) techniques (Johannsen et al., 2000; Rodell et al., 2012). Brain functional decline may result from different pathogenetic processes in different forms of dementia, however. Damage to dendritic spines with impairment of the coupling of flow and metabolism to the excitation of post-synaptic neurons may be common to processes of brain functional decline in dementia (Penzes et al., 2011).

Alzheimer’s disease (AD) is the most common type of dementia, afflicting more than 20 million victims worldwide. The definitive diagnosis of AD requires post-mortem identification of senile plaques of amyloid-beta (Aβ) protein and neurofibrillary tangles of tau protein (McKhann et al., 1984; Dubois et al., 2007), but the etiology of AD is poorly understood, and the causative role of senile plaques is debated.

The amyloid cascade hypothesis claims that erroneous cleavage of the amyloid precursor protein (APP) generates Aβ that accumulates as senile plaques, associated with damage to dendritic spines and loss of functional connectivity (Hardy and Higgins, 1992; Hardy and Selkoe, 2002, Harrison and Owen, 2016). The resulting dementia of Alzheimer’s type is associated with degeneration of specific functional networks, and regionally differentiated degrees of amyloid deposition that may explain part of the clinical heterogeneity of symptoms (Lehmann et al., 2013) and hence may be important to diagnosis and prognosis.

Of the multiple potential paths to functional decline, amyloid deposition is not the exclusive marker, with markers of other pathogenetic processes suggesting paths to other types of dementia, such as Lewy Body Dementia (DLB) or frontotemporal lobar degeneration (FTLD). The diversity of pathologies underlying the functional decline in dementia raises the need for comparative evidence from functional and pathological markers that distinguish among the different types of dementia. In these disorders, the relationship between the pathological changes and the changes of brain energy metabolism and blood flow is not fully understood. An approapriate approach would assess both markers of brain pathology and functional integrity.

The pathology associated with specific types of dementia, notably AD, can be detected by means of a specific marker of the presence of Aβ, by means of Pittsburgh Compound B, [^11^C]PiB, a tracer used with PET (Cohen et al., 2012), or widespread clinically with [^18^F]-labeled amyloid PET tracers. While [^11^C]PiB is useful in the differential diagnosis between AD and FTLD, it is not alone useful in differentiating AD from DLB, where 70–80% of the cases present with Aβ pathology.

Recently, we exploited a particular property of the brain uptake of [^11^C]PiB to show that it is possible to simultaneously extract a functional index in terms of an absolute measure of cerebral blood flow, as well as a pathological index in terms of the absolute degree of binding of [^11^C]PiB in brain tissue, both obtained with the Washout Allometric Reference Method (WARM; Rodell et al., 2013). By application of WARM, we showed that it is possible to use maps of brain uptake of [^11^C]PiB to simultaneously determine a surrogate measure of absolute cerebral blood flow and a measure of amyloid-beta accumulation in brain. The quantitation depends on the rapid initial clearance of [^11^C]PiB from the circulation, which is consistent with the initial flow-limited uptake of [^11^C]PiB, such that kinetic analyses of the uptake and binding take both blood flow and washout into account in a manner that couples Aβ plaque load to the cerebral blood flow (Johannsen et al., 2000), including the loss of flow variability observed in AD (Rodell et al., 2012).

Given the novel WARM-based identification of both [^11^C]PiB binding, estimated from the rate of tracer washout as an index of amyloid load, and an index of blood flow, estimated from the intitial flow-dependent [^11^C]PiB brain distribution as a marker of functional activity, we here test the hypothesis that the degree of correlation between plaque load and the blood flow index accurately differentiates among the four different forms of dementia or degeneration examined here.

## Materials and Methods

The flow-independent WARM (Rodell et al., 2013) addresses both the flow-dependent accumulation and the rapid clearance of [^11^C]PiB from the circulation. Here, we evaluate the estimates of the two variables generated by WARM, in order to determine the usefulness of each as a diagnostic of dementia, and specifically of DAT. We weighed the estimates against the conventional Standard Uptake Value Ratio (SUVR). In order to make the analysis independent of a specific threshold, we report the Receiver Operating Characteristic (ROC) curves for each method, as calculated from a data set of subjects with different dementias and healthy age-matched volunteers.

### Subjects

Twenty-nine aged individuals with well-documented normal cognitive function (HC), 16 patients with mild to moderate AD, 8 patients with DLB, 5 patients with FTLD, and 5 subjects with MCI, previously described by Rowe et al. (2007), were re-analyzed for flow-independent *BP*_ND_ and sCBF estimates, using WARM (Rodell et al., 2013). Normal volunteers originally recruited from a cohort of subjects participating in the longitudinal Healthy Aging Study at the Mental Health Research Institute of Victoria were shown to have normal cognitive performance on neuropsychological testing that included California Verbal Learning Test (CVLT II), Rey figure, Logical Memory, verbal and categorical fluency, Boston Naming Task, and digit span. All subjects in the current study were assessed with this neuropsychological test battery, the Mini-Mental State Examination (MMSE), and the Clinical Dementia Rating within 1 week of the PiB PET acquisition. All AD patients met National Institute of Neurological and Communication Disorders and Stroke and the AD and Related Disorders Association criteria for probable Alzheimer’s disease (pAD), whereas all subjects in the DLB group met the consensus criteria for probable DLB of cognitive fluctuation, visual hallucinations, and parkinsonism. FTLD subjects had characteristic clinical presentation and frontal lobe or temporal lobe atrophy on MRI with concordant hypometabolism on fluorodeoxyglucose PET. The MCI subjects met the Petersen criteria of subjective and objective cognitive difficulties, predominantly affecting memory, in the absence of dementia or significant functional loss (Petersen et al., 1999). All patients were recruited from the Austin Health Memory Disorders and Neurobehavioral Clinics, and further details can be found in Rowe et al. (2007). The SUVR measures of [^11^C]PiB retention previously were published for subjects and healthy volunteers used in the present study (Rowe et al., 2007) while *BP*_ND_ and sCBF measures were not previously obtained or reported.

### Positron Emission Tomography

#### Image Acquisition

All subjects had dynamic [^11^C]PiB PET acquisitions. [^11^C]PiB was produced at Centre for PET, Austin Hospital, using the one-step [^11^C]methyltriflate approach (Wilson et al., 2004) The average radiochemical yield was 30% after 45 min synthesis with radiochemical purity of >98% and specific activity of 30 ± 7.5 GBq/μmol. Each subject received 375 MBq [^11^C]PiB by IV injection over 1 min. PET acquisitions were carried out on a Phillips Allegro PET device. Rotation transmission sinogram acquisition in three-dimensional mode was made with a single [^137^Cs] point source before the injection of the radiotracer for attenuation correction. A 90-min list-mode emission acquisition was performed in three-dimensional mode after injection of [^11^C]PiB. List-mode raw data were sorted off line into 4 × 30-s, 9 × 1-min, 3 × 3-min, 10 × 6-min, and 2 × 10-min frames. The sorted sinograms were reconstructed using a three-dimensional RAMLA algorithm.

#### Image Registration and Segmentation

Summed emission scans of [^11^C]PiB uptake were automatically co-registered to a standardized mean [^11^C]PiB image in common stereotactic space (ICBM, Montreal Neurological Institute; Mazziotta et al., 2001) using a six-parameter affine transformation. We did not use individual MR anatomical images. After calculation of the final linear PET-Talairach transformations, dynamic emission scans were re-sampled into common coordinates. Regional *BP*_ND_, *R*_1_, SUVR, and sCBF measures (see below) were obtained from parametric PET image maps using standard modelbased segmentation (Collins et al., 1994; Grabner et al., 2006). The regions analyzed included left and right frontal (FL), occipital (OL), parietal (PL), and temporal (TL) lobes, as well as white matter (WM) and the cerebellar grey matter (CERB).

### Quantification of [^11^C]PiB Variables

#### Reference Region Ratio Measure (SUVR)

[^11^C]PiB retention can be calculated by determining the accumulation relative to a reference tissue, to obtain a ratio measure (SUVR). The ratio measure is the fraction of the region-of-interest integral of [^11^C]PiB accumulation at steady-state, assumed to have been established no later than this time after injection (*t*_s_ = 40 min), extended to the end (*t*_e_ = 60 min), relative to the integral of the [^11^C]PiB accumulation observed in the same period in the reference region. In the reference region, the accumulated tracer as function of time, *m*_ND_(t), represents the non-specific bound tracer after delivery by homogeneous flow to all voxels of the reference region. We further assumed the interval from *t*_s_ = 40 min to *t*_e_ = 60 min to be sufficient to establish steady-state (secular equilibrium) in all regions, as required by the definition of the volume of distribution of the tracer, *V*_T_, as the sum of the volumes of distribution of non-displaceable tracer (*V*_ND_) and the additional volume of distribution of reversibly bound tracer.

#### Washout Allometric Reference Method (WARM)

In the case of negligible contribution from the circulation after the initial brief uptake, the tissue time-activity curves of the radio ligand are established by the radioactivity initially dispersed to the tissue and the subsequent washout from the brain regions of uptake. WARM (Rodell et al., 2013) specifically takes this property into account and uses only the differences among washout rates from regions with different properties of binding, blood flow rates, and blood-brain barrier permeability to determine the binding of the tracer. For direct calculation without linearization, *BP*_ND_ can be found using the operational equation (Rodell et al., 2013),

$${BP}_{ND}(T)=\frac{m(0)\int_{0}^{T}((In\left(m_{ND}\left(t\right)\right))-\left(In\left(m_{ND}\left(0\right)\right)\right))dt}{m_{ND}(0)\int_{0}^{T}((In\left(m\left(t\right)\right))-\left(In\left(m\left(0\right)\right)\right))dt}-1$$

where *m*_ND_(t) represents the non-specifically bound tracer following delivery by the circulation, and *m*(t) represents the tracer in any other region as function of time *t*, where *T* represents the specific time *T* after injection of the tracer. Due to the rapid clearance of the tracer from the circulation, analysis of the dynamic [^11^C]PiB image data revealed an initial high-frequency signal of arterial origin in the first 2 min of acquisition, followed first by a peak within the time frame 2–10 min, from which time washout prevailed. For WARM, we split the signal into the three time frames of, first, arterial phase, second, peak uptake, and third, washout. We used the peak value within the 2–10 min interval for estimation of the relative uptake coefficient *R*_PiB_ relative to the cerebellum grey matter value. In order to calculate the calibrated sCBF, we estimated the slope of the rise to the peak as the ratio of the peak value to the time spent to reach the peak (peak value/time to peak) of the [^11^C]PiB signal.

We used the phase of washout from the peak in the time interval 2–60 min of dynamic image data for estimation of the binding potential *BP*_ND_ (Rodell et al., 2013). *BP*_ND_ is is derived in Eq. (1). When log-transformed, in the fraction part of the equation, the nominator is the accumulated log-signal for the reference tissue, relative to the tracer present before washout (i.e., the tracer that is left, compared to the tracer initially deposited in the reference region). We scaled this difference by the initial tracer uptake into the ROI region. The denominator similarly describes the accumulated log-signal for a ROI or voxel relative to how much was initially present before washout. This difference is scaled by the start amount of the reference region. Thus the fraction is corrected both for flow, i.e., initially deposited tracer, and for the exponential behavior of the washout (Rodell et al., 2013).

In order to test the power of WARM to distinguish the dementia (AD, DLB, FTLD) and, MCI from the HC group by quantification of the amount of retention, we extracted regional parametric values for the [^11^C]PiB retention from the parametric images with the different approaches of WARM and SUVR. For WARM, we reported mean absolute *BP*_ND_ values, and for SUVR we extracted fractional values of the area under the curves (40–60 min), relative to the cerebelum gray matter values. As no calibration values of sCBF from PET measures based on [^15^O]water were available for this data set, we used a site-specific scale factor to obtain a simulated absolute flow estimate from the peak tracer arrival characteristics. The factor was derived as an offset for the blinded data set, with a target of the corresponding mean CBF value reported by Rodell et al. (2013). While the use of a scaling factor may not yield absolute flow measures, the test-specific values are stable as intrasubject comparisons when afhered to a particular site and protocol, also without a [^15^O]water calibration, in contrast to the fractional *R*_PiB_ values. We used the scaling factor in order to facilitate comparison to the findings reported by Rodell et al. (2013).

### Receiver Operating Characteristics

Receiver operating characteristic curves define a cutoff value for clinical testing (Senanarong et al., 2012). Here, we determined the test threshold as the coordinates of the point of the ROC curve closest to the upper left corner of the ROC curve. We obtained an additional important measure of the accuracy of the clinical test as the area under the ROC curve. This discrimination is reliable when both sensitivity and specificity are close to 1.0, with minimal false positive and negative findings.

## Results

### Separate Brain Images from the Two Phases of Tracer Kinetics

**FIGURE 1:**
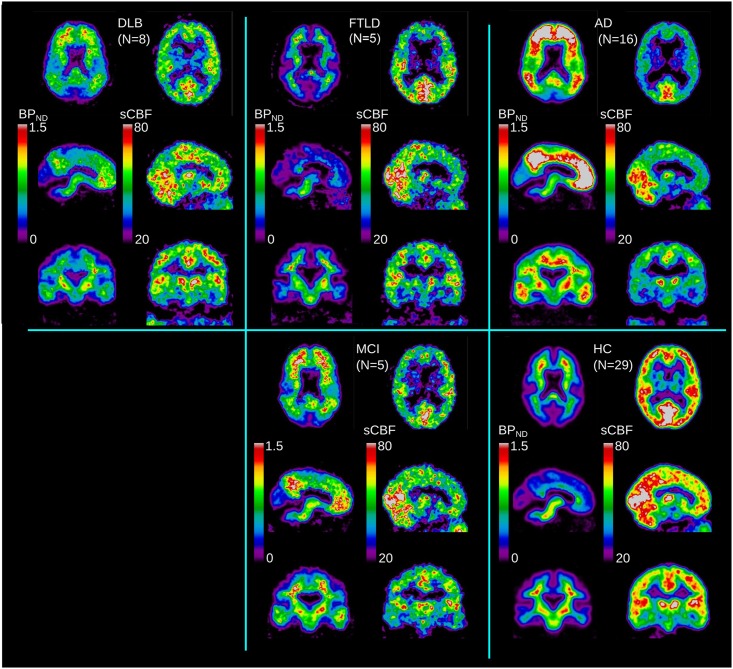
**Average images for each diagnostic group (DLB, AD, FTLD, MCI) and HC, for binding potential (*BP*_ND_) and *sCBF* measures**.

Due to the rapid clearance of the tracer from the circulation, the dynamic [^11^C]PiB image data revealed an initial high-frequency signal of arterial origin in the first 2 min, consistent with a peak within the 2–10 min interval, followed by the washout phase. For the application of WARM, we split the signal into three time intervals of arterial phase, peak uptake phase, and washout phase. We used the maximum peak within the 2–10 min peak uptake phase to estimate the relative uptake coefficient *R*_1_ and the calibrated surrogate CBF index (sCBF) directly from the [^11^C]PiB signal. We used the washout phase from the maximum of the peak phase within the 2–60 min dynamic acquisition to estimate the binding potential *BP*_ND_ of [^11^C]PiB. The two derived images are then defined as the sCBF image obtained from the initial uptake, and the *BP*_ND_ image obtained from the washout part when the tracer deposit is complete and washout occurs relative to the initial deposit. **Figure 1** presents the two time-averaged images of sCBF and *BP*_ND_ for each of the groups.

### High Amyloid Deposition and Decreased Blood Flow in AD

For AD, we confirmed very significant (*P* < 0.0001) increases of [^11^C]PiB binding by both the SUVR and *BP*_ND_ measures in the FL, PL, TL, and OL regions, and for the SUVR measure also a significant (*P* < 0.01) difference in the reference region where we had predicted no specific binding. The *BP*_ND_ measure revealed increased binding in WM, assumed to represent radioactivity spillover from the significant cortical binding in general. Examination of individual contributions revealed less binding estimated as *BP*_ND_ in the OL region than in the FL, TL, and PL regions in every individual. Three individuals with markedly high binding in the OL region also revealed the highest retention in the remaining regions, suggesting that the OL region might be the last region to be affected by amyloid deposition. Although the binding in the AD patients was the more significant measure, the group as a whole had marked decrease of the sCBF estimate, particularly in FL and PL.

**FIGURE 2:**
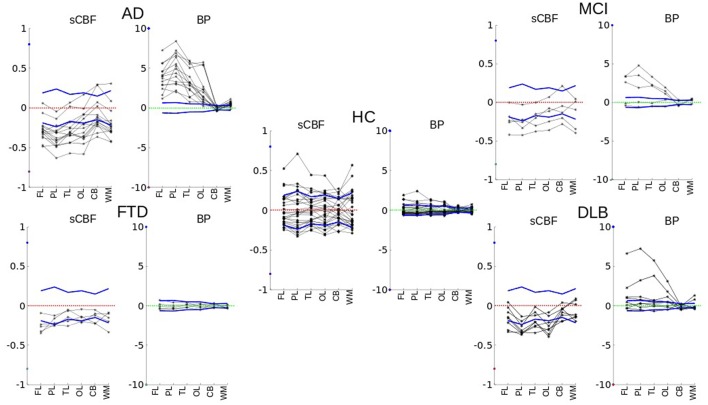
**Single individual subject values vary in relation to the normal variation of the members of the HC group, for each region (FL, PL, TL, OL, CB, WM) of each subject.** Both sCBF and *BP*_ND_ variability values for different cortical regions and white matter are plotted for each diagnostic group. The figure shows how values differ relative to the mean of healthy controls. The dotted zero-lines represent the normalized averages of the healthy control subjects. Solid blue lines are normalized standard deviations of the group of healthy volunteers.

**FIGURE 3:**
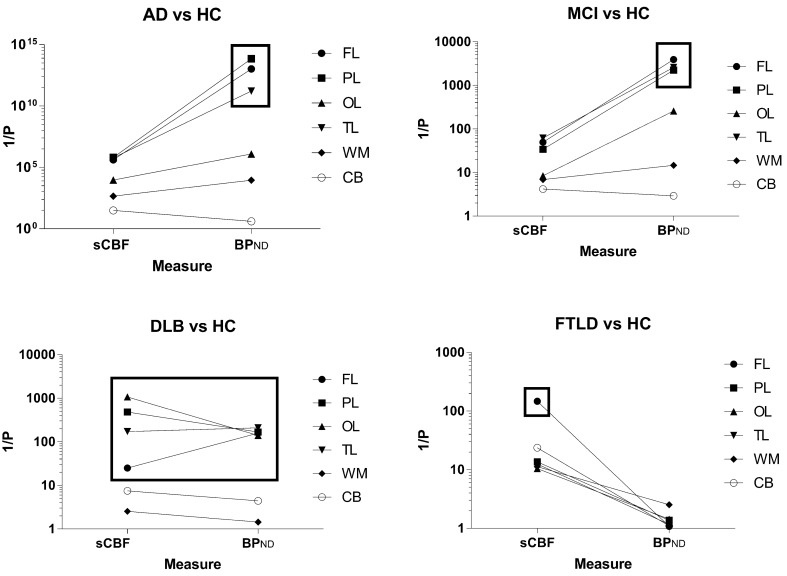
**The reciprocal of the statistical significance measure P (obtained by the Student *t*-test) of each diagnostic group versus HC for each parameter.** The graphs indicate the importance of each of the two parameters for identification of the disease group in comparison to the healthy control subjects. The cerebellum gray matter was used as reference for the binding calculation, and results for this region are depicted with the open circles.

In the MCI category, *BP*_ND_ estimates were significantly higher (*P* < 0.001), with three of the five patients having AD-like tracer retention in the PL, TL, FL, and OL regions as shown in **Figure 2** (MCI). As seen in **Figure 3**, the *BP*_ND_ measure was an order of magnitude higher than for the SUVR measure. Of all cortical lobes, the OL region was the least affected. This pattern was similar for the sCBF measure that was not significantly different in the OL region but was significantly lower (*P* < 0.05) in the TL, Pl, and FL regions.

### Low Amyloid Deposition and Decreased Blood Flow in DLB

Only two of the eight patients diagnosed with DLB had high [^11^C]PiB retention, as shown in **Figure 2** (DLB), sufficient to distinguish the group as a whole from the HC for the FL, PL, TL, and OL regions. The *BP*_ND_ obtained with WARM distinguished the group more significantly from HC than the SUVR measure, as shown in **Figure 3**. In contrast, the sCBF measure revealed very significantly reduced blood flow compared to healthy volunteers. The decrease was most pronounced (*P* < 0.001) for the OL region, followed by the PL region (*P*∼ 0.002), while the TL (*P* < 0.01) and FL (*P* < 0.05) regions had less markedly reduced blood flow. The decrease in this group is is in contrast to the finindgs in the AD and MCI groups, in which the OL region remained the least affected of the cortical lobes.

### No Amyloid Deposition and Markedly Reduced Frontal Lobe Blood Flow in FTLD

Patients with FTLD had low [^11^C]PiB retention when compared to the HC group, expressed as the binding potential *BP*_ND_, in agreement with findings previously reported as SUVR by Rowe et al. (2007). In contrast, this small group of patients had significantly reduced blood flow measured as sCBF in the FL region (*P* < 0.007) and cerebellum (*P* < 0.05), presented in **Figures 2** and **3**. When normalized to cerebellum, the decrease of sCBF in FL remained significant. The SUVR, measure indicated significant increases in the WM, but the *BP*_ND_ measure by WARM did not confirm the increase.

### Correlations between *BP*_ND_ and sCBF Measures

**FIGURE 4:**
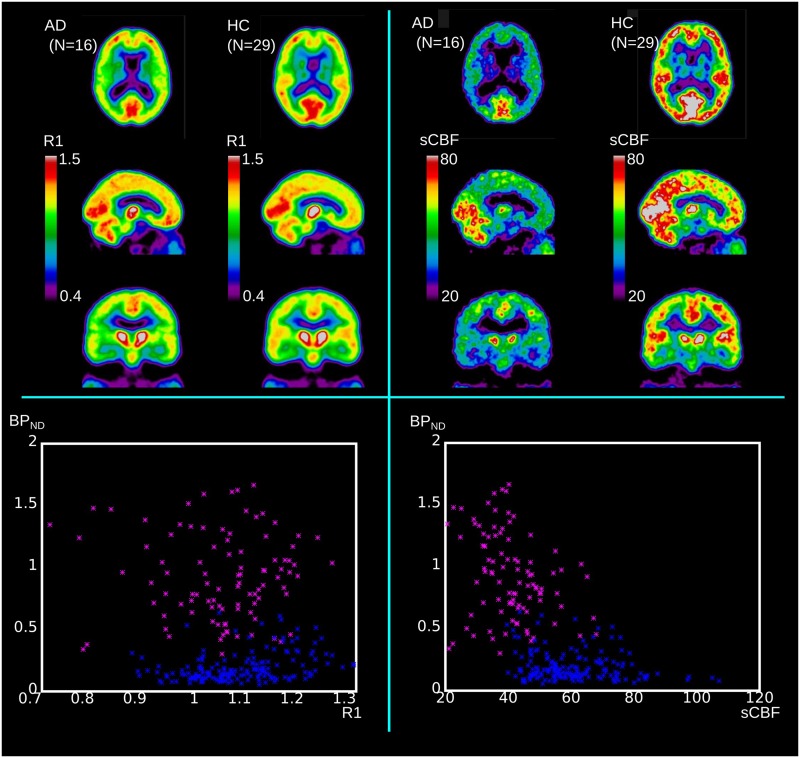
**Upper panels show mean values of *R*_1_ and sCBF for comparison of AD to HC groups.** Lower panels illustrate how relative *R*_1_ and absolute sCBF measures differ in relation to *BP*_ND_ measures. Blue dots represent HC regional values (unilateral values of FL, PL, TL, and OL) and magenta dots represent similar regional values for AD subjects.

While all dementia groups on average revealed lower cortical blood flow regardless of diagnosis, only the AD and MCI groups had unequivocal correlations with [^11^C]PiB retention, expressed as the tracer *BP*_ND_ and sCBF measures, respectively. **Figure 4** shows the mean images of the *R*_1_ and sCBF values for the AD and HC groups, as well as the distinguishing relations of the *R*_1_ and sCBF estimates to the *BP*_ND_ binding measures for unilateral values in the FL, PL, TL, and OL regions of each subject. The *BP*_ND_ measure was inversely related to the sCBF measure in AD, but the relative flow measure *R*_1_ had no correlation.

Of the eight DLB subjects, two had very high values of cortical [^11^C]PiB binding, but only one had a marked decrease of the sCBF estimate, compared to the healthy control condition. Three DLB subjects had moderate binding of [^11^C]PiB together with decreased sCBF values, while two had normal cortical *BP*_ND_ values as well as slightly decreased sCBF values, as illustrated in **Figure 2** (DLB).

The images of members of the FTLD group were interpreted as normal with respect to [^11^C]PiB binding, but they all had marked frontal sCBF deficits, as illustrated in **Figures 2** and **3**. The deficits were also visible on the relative (*R*_1_) flow images (not shown). The [^11^C]PiB binding and sCBF indices in cerebellum and occipital cortex appeared unaffected by FTLD.

As 60% of the MCI patients had the typical AD pattern with high cortical [^11^C]PiB binding and low flow, the group result is comparable to the AD but with less marked findings, as illustrated in **Figures 2** and **3**.

### Receiver Operating Characteristics

**FIGURE 5:**
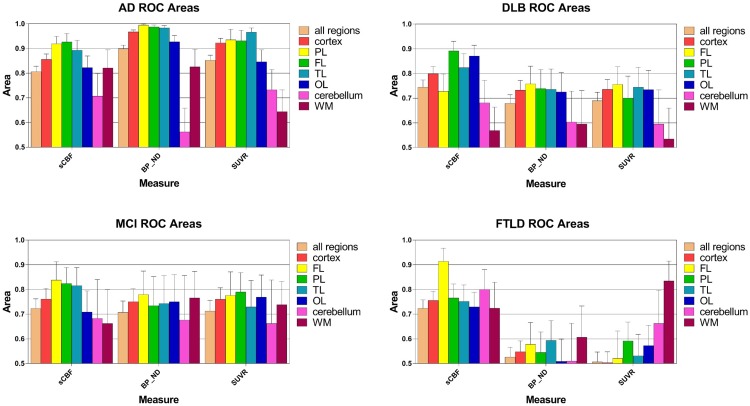
**Areas under receiver-operating curves (ROC) for all regions, ±SEM, including cortex, frontal (FL), parietal (PL), temporal (TL), and occipital (OL) lobes as well as cerebellum gray matter and white matter (WM).** Each separate graph represents a single diagnostic group (AD, DLB, MCI, FTLD). The integral of the ROC curve is plotted for each variable. Areas close to unity represent a high level of true positives and low level of false negatives, i.e., higher diagnostic certainty. Areas close to 0.5 means that the ROC curve is close to the line of unity at which there is no difference among the distributions of values in the pathological condition and in the group of the HC, i.e., least disease discrimination, as expected for the reference region.

**Figure 5** shows the areas under the ROC curves for all cortical regions, as well as cerebellum gray matter and WM. Separate plots represent a separate diagnostic group, with the integral of the ROC curve plotted for each of them. Areas close to unity represent high incidences of true positives and low incidences of false negatives, consistent with higher diagnostic accuracy. Areas close to 0.5 indicate ROC curves close to the line of identity at which there is no difference between the distributions of the values of patients and healthy volunteers, i.e., a minimum of diagnostic discrimination, as expected of a true reference region.

AUC measures are shown in **Figure 5**. For the AD group of subjects, ROC areas for the cortex implied that *BP*_ND_ estimates determined with WARM had the highest power of discrimination among patients and healthy volunteers, followed by the SUVR and sCBF but only the *BP*_ND_ estimates were significantly different (*P* < 0.05) from sCBF estimates in pairwise ROC comparisons of the three methods (DeLong et al., 1988). Interestingly, only the *BP*_ND_ measures were significantly superior (*P* < 0.05) determinants to the MMSE scores (data not shown). For the MCI group, the ROC areas were not significantly different for the SUVR and *BP*_ND_ estimates, but somewhat higher for the FL, PL, and TL regions. However, for the FL region, the sCBF estimates as determinants were significantly superior to the SUVR estimates (*P* < 0.05).

For the DLB group of subjects, the measures that most significantly distinguished the patients from the HC group of subjects included declines of the sCBF values in the CORTEX (*P* < 0.05 compared to *BP*_ND_) and PL and TL (*P* < 0.05 compared to *BP*_ND_ and SUVR) regions, in contrast to the MCI and AD groups of subjects in whom flow declines in FL and [^11^C]PiB retention in the PL, TL, and FL regions were more distinct than in the OL region, although all three measures for the AD group of subjects were not significantly different in single regions alone. Also for the AD and MCI groups of subjects, the sCBF measure in the OL region was less affected than in the PL, TL, and FL regions. For FTLD, the ROC area of the sCBF measure was a significantly superior (*P* < 0.05) discriminator compared to the SUVR measure of the FL only.

## Discussion

The novel WARM analysis (Rodell et al., 2013) yields quantitative measures both of the surrogate absolute blood flow value and of the binding potential of the amyloid-β-avid tracer [^11^C]PiB. The aim of this study was to evaluate the power of the analysis to distinguish among and between aging and different diagnostic groups associated with neurodegenerative disorders (Fast et al., 2013; Gjedde et al., 2013). The major findings of the study include the delineation of the pathophysiology of the individual diagnostic groups along the two orthogonal axes of the measure of Aβ deposits and the measure of cerebral blood flow. This property of WARM allowed us to distinguish among the different forms of dementia by two-dimensional plotting of the results. The distinctive patterns were evident from the relationships among the magnitudes of functional decline, identified from the measure of blood flow, and the degree of pathology, identified from the measure of amyloid-β load, both obtained from a single dynamic PET imaging session.

We determined the two key measures from different temporal intervals of the PiB uptake dynamics. The two measures included the sCBF, the patient-specific blood flow measure that is decisively different from the relative perfusion measure *R*_1_, and the binding potential *BP*_ND_, both revealed by WARM. All dementia groups, regardless of diagnosis, had significantly lower cortical flow, expressed as sCBF than the healthy volunteers, while only the AD and MCI diagnostic groups had unequivocally elevated [^11^C]PiB retention, expressed as the binding potential of the tracer (*BP*_ND_).

Importantly, this observation implies that the reduction of blood flow is the functionally relevant measure, while the elevated *BP*_ND_ is the result of an etiologically specific pathological process of AD and MCI on one hand, and of DLB on the other. In the AD and MCI groups, the *BP*_ND_ measure discriminated the patients from the HC group to a greater extent than the conventional late-timepoint SUVR measure, in part because of the flow sensitivity of the SUVR measure, as apparent from the WM binding of healthy controls.

Three different patterns of relative change of the two chief measures distinguished the diagnostic group members from the healthy control subjects, as illustrated in **Figure 3**. The FTLD and DLB group members had significantly reduced flow (FTLD), or significantly reduced flow with equally raised binding (DLB), respectively. The AD and MCI group members had significantly increased binding with much smaller degrees of flow reduction. The MCI group members were characterized by the primary but less pronounced increased binding of the tracer, compared to the AD group members. The binding in the AD patients was the more significant measure, but the group as a whole had marked decrease of the sCBF estimates, particularly in FL and PL, as previously reported by Johannsen et al. (2000). This finding is of considerable interest because a matching decline of glucose metabolism generally is not present (Edison et al., 2007), although lowered metabolism generally is evident also in FL in addition to the pathognomonic decline in parietal lobe (Gejl et al., 2012, 2013, 2014, 2016). The low flow may be a physiological reaction to the functional decline of the diseased brain, as oxygen delivery is commensurate with the reduced consumption of oxygen (Rodell et al., 2012). More or less normal glucose consumption therefore implies that a greater faction of the glucose undergoes aerobic glycolysis only.

The [^11^C]PiB-derived sCBF deficits in the frontal lobe distinguished FTLD patients from the members of the HC group that were otherwise indistinguishable with respect to the binding of [^11^C]PiB. The combination of the two measures therefore improves the ability of [^11^C]PIB to discriminate between AD (including MCI), DLB, and FTLD. A link between Aβ pathology and functional decline in AD and MCI might be the most likely reason for the high correlation between the flow and binding measures in AD. The different correlations distinguished accurately among the remaining diagnostic groups. Although based on a small number of subjects, the finding has implications for future combinations of other absolute flow measures using PET or MR techniques with amyloid PET imaging, also for the more clinically applicable [^18^F]-labeled tracers.

The use of WARM to derive multiparametric measures from a single dynamic dataset more clearly differentiates among functional and pathological features than the SUVR measure for the individuals analysed in the present study. The study also demonstrates that care must be taken to interpret relative measures such as *R*_1_ with caution, as they had no determining power, compared to the subject specific quantitative measure of sCBF in this study (Borghammer et al., 2009a,b). The two variable approach is promising for other PET tracers where the rapid disappearance from the blood stream decouples the inflow and outflow phases of the kinetics.

## EthIcs Statement

Approval for the study was obtained from the Austin Health Human Research Ethics Committee. All patients were recruited from the Austin Health Memory Disorders and Neurobehavioral Clinics. Written informed consent for participation in this study was obtained prior to the scan.

## Author Contributions

AR and AG: Did the data-analysis, method development, and manuscript preparation. VV, CR and GO: Did the recruiting, data-collection, and manuscript preparation.

## Conflict of Interest Statement

The authors declare that the research was conducted in the absence of any commercial or financial relationships that could be construed as a potential conflict of interest.
